# Phentermine–topiramate extended release for the dual treatment of obesity and sleep-related eating disorder: a case report

**DOI:** 10.1186/s13256-021-03250-1

**Published:** 2022-01-27

**Authors:** Eduardo Grunvald, Jennifer DeConde

**Affiliations:** 1 Division of General Internal Medicine, Department of Medicine, University of California San Diego, San Diego, CA USA; 2 Bariatric and Metabolic Institute, Division of Minimally Invasive Surgery, Department of Surgery, University of California San Diego, 4303 La Jolla Village Drive, Suite 2110, San Diego, CA 92122 USA

**Keywords:** Obesity, Eating disorders, Sleep-related eating disorder, Topiramate, Phentermine, Case report

## Abstract

**Background:**

Obesity and eating disorders can present together, and pose diagnostic and therapeutic challenges to the clinician. Generally, lifestyle interventions alone for the treatment of obesity have modest long-term effectiveness. Phentermine–topiramate extended release is a relatively new medication approved for weight reduction. Sleep-related eating disorder is a rare condition that is often underdiagnosed. Both conditions are chronic and require long-term management. There is no definitive treatment for sleep-related eating disorder, and therapeutic options are based on case reports.

**Case presentation:**

A 35-year-old Caucasian male with a body mass index of 41.7 kg/m^2^ presented for obesity treatment. History revealed nocturnal episodes of hyperphagia associated with amnesia of overeating and other features of sleep-related eating disorder. Treatment was initiated with phentermine–topiramate extended release. Five months later he lost 5% of his body weight and demonstrated resolution of sleep-related eating disorder behaviors. He reported no adverse side effects. Upon self-discontinuation of the medication, his eating disorder recurred.

**Conclusions:**

Clinicians intending to help patients reduce body weight should screen for nocturnal eating and other eating disorders. Sleep-related eating disorder can be associated with significant morbidity and excess weight. Patients report adverse effects on quality of life as a result. Phentermine–topiramate extended release may be a good therapeutic option for patients presenting with comorbid obesity and sleep-related eating disorder. More research is needed to explore the efficacy and safety in this patient population.

## Introduction

Obesity is a complex disease with multiple contributing factors and great etiological heterogeneity among individuals. In some, eating disorders may play a major role. There is significant overlap between binge eating disorder (BED) and night eating syndrome (NES) with overweight and obesity [1]. Comorbid eating disorders are sometimes overlooked during assessment and management of weight reduction treatment strategies. Sleep-related eating disorder (SRED) is an uncommon condition that can contribute to disordered eating and overconsumption of calories. Topiramate has been reported to improve nocturnal eating episodes and has weight reducing—or leptogenic—properties. Phentermine is a commonly prescribed adrenergic agent for the treatment of obesity. These two drugs have been combined with an extended release formulation as an anti-obesity medication (AOM), approved by the United States Food and Drug Administration (US FDA). Since obesity is considered a chronic metabolic disease [2], this agent is approved for long-term use.

Here we present a case of a patient with obesity and SRED treated with phentermine–topiramate extended release (PHEN-TPM ER), resulting in the dual benefit of weight loss and resolution of pathologic hyperphagia at night. This report underlines the importance of screening for eating disorders in patients with obesity, presents the clinical features of SRED, reviews the pharmacologic management of nocturnal eating syndromes, and exemplifies the use of a novel AOM for patients with obesity and SRED.

## Case presentation

A 35-year-old Caucasian male was referred to our comprehensive obesity clinic for medical weight management. He presented with a past medical history of fatty liver disease, hypertension, and hypothyroidism. He denied insomnia or mood disturbances. He drank 4–5 alcoholic drinks per day on the weekends. He was a former smoker but denied illicit drug use. His diet was high in processed carbohydrates and saturated fats, and he did not engage in any regular physical activity. He was unable to maintain sustained weight loss, with several self-directed interventions in the past. Medications included amlodipine, hydrochlorothiazide, lisinopril, and levothyroxine. He did not use any agents for sleep or psychiatric indications. Blood pressure was 132/84 mm Hg, heart rate 86 beats per minute, oxygen saturation 97% on room air, weight 135.7 kg, with a body mass index (BMI) of 41.7 kg/m^2^. Physical examination was notable for a waist circumference of 136 cm and a narrow airway with Mallampati score of III.

The patient reported that on most nights, for several years, he would consume food in the kitchen or bedroom without awareness and with complete amnesia of the behavior when he awoke in the morning. On occasion, he noticed particles of food or wrappers in the bed. He reported morning anorexia. There were no associated injuries. There was no history of purging.

In addition to engaging in nutrition modification, physical activity, and behavior therapy, adjunctive pharmacotherapy with PHEN-TPM ER was initiated, titrated to 7.5–46 mg daily. He was also counseled on reducing alcohol consumption. Two months later, he had lost 6.8 kg (5% of initial weight), with complete cessation of night eating episodes and marked reduction of alcohol consumption. At follow-up 3 months later, he had maintained 5% weight loss despite intermittent use of the medication (due to insurance authorization challenges) and fragmented adherence to lifestyle changes. An overnight video polysomnogram performed 3 months after presentation while taking PHEN-TPM ER revealed moderate obstructive sleep apnea (OSA) with no significant periodic limb movements nor observed somnambulism. Apnea–hypopnea index was 27 events per hour and oxygen saturation nadir was 87%.

The patient was then lost to follow-up and returned 6 months later, having self-discontinued PHEN-TPM ER. He had maintained his weight loss, but his night-eating behaviors had recurred. He reported poor adherence to positive pressure therapy due to discomfort from the nasal mask. Having achieved remission of nocturnal eating behaviors, he expressed interest in resuming the medication more for that purpose than weight loss. The patient endorsed that resolution of his nocturnal binges had great benefit on quality of life.

## Discussion

Here we present a case of a patient with obesity and SRED that responded promptly to standard dose PHEN-TPM ER. Obesity is a complex disease with a broad spectrum of contributing factors, each with its own relative impact. Eating disorders, particularly BED, are associated with increased BMI [3]. NES, a less common condition, has also been linked to weight gain [4]. SRED is often underdiagnosed and characterized by recurrent episodes of compulsive eating during arousal from sleep, with partial or complete amnesia of the event. The text box lists the diagnostic criteria [5]. Eating usually occurs in an uncontrollable manner, with a predilection for high-calorie foods or even toxic or inedible items. Patients often report nonrestorative sleep with resulting daytime fatigue and morning anorexia. Sleep-related injury can occur, such as cutting oneself while preparing food, burns, or falls. A downstream adverse effect is undesirable weight gain from recurrent binge eating. SRED can be associated with other sleep disorders, including sleepwalking, restless leg syndrome (RLS), narcolepsy type 1, and OSA. It has also been associated with hypnotic and psychotropic medication use, including zolpidem, tricyclic antidepressants, anticholinergics, lithium, and antipsychotics [6]. There may be comorbid mood disorders or recent life stressors. Women are more affected than men, and age of onset is usually in late adolescence or early adulthood. There is some evidence that SRED may have a genetic predisposition. The incidence of SRED in the general population is unknown, but one analysis has estimated the incidence at 1% among individuals seeking treatment for obesity [7].

Based on case reports, treatment modalities include topiramate, selective serotonin reuptake inhibitors, benzodiazepines, or dopamine agonists if there is associated RLS [8]. All pharmacologic therapies are considered off-label and successful treatment can be challenging. Management of SRED with topiramate, an anticonvulsant used for seizure disorders and migraine headaches, is reported in the literature [9]. Not approved by the FDA for treatment of SRED, topiramate has also been used successfully for NES [10]. Although its use is off-label as anti-obesity pharmacotherapy, randomized controlled trials have shown efficacy for weight reduction [11–13]. As previously noted, it is one of the components in the FDA-approved AOM, PHEN-TPM ER. Two-year data has shown a mean weight loss of 10.7%, compared with 2.2% in the placebo group [14].

It is not completely clear how topiramate induces weight loss, but animal studies have shown its leptogenic effects potentially through central nervous system (CNS)-mediated suppression of energy consumption and a reduction of energy utilization efficiency through peripheral thermogenic mechanisms [12]. It is unknown how topiramate may be therapeutic for SRED, but it has been posited that it may have anorexigenic effects and suppress arousal [10].

PHEN-TPM ER is FDA-approved for the treatment of overweight and obesity and contains a combination of phentermine with topiramate in a controlled release formulation. The leptogenic properties of phentermine are due to increased norepinephrine levels in various relevant brain structures. Combining the two agents allows for enhanced efficacy through targeting several pathways in the CNS, while limiting adverse effects by utilizing lower doses of each component. The most common dose of phentermine monotherapy used by many providers is 37.5 mg daily, in contrast to 7.5 mg daily in the standard formulation of PHEN-TPM ER, a fifth of the dose. Even for the highest strength, 15 mg of phentermine and 92 mg of topiramate, the doses are lower than those often given with either monotherapy alone. The anorexigenic effects of both phentermine and topiramate may be synergistic for the therapy of SRED. Moreover, since both obesity and SRED are chronic conditions, they require ongoing therapy, and relapse would be expected with cessation of treatment, as exemplified with the patient in this report. Because of this understanding, the newest generation of AOMs, including PHEN-TPM ER, are approved by the FDA for long-term use.

Since unrecognized SRED may be a significant driver of elevated BMI, individuals with obesity should be screened for these behaviors, among other eating disorders. Challenges for clinics focused on treating obesity include time constraints, high patient volume, lack of knowledge regarding criteria for various eating disorders, feature overlap between eating disorders and overeating in common obesity, and the internal biases and embarrassment of patients leading to underreporting of specific signs and symptoms. The clinician should, therefore, have a high level of suspicion and at least screen all individuals with overweight or obesity presenting for evaluation and treatment. In the primary care setting, many of these challenges are amplified due to contemporary health care delivery pressures. Obtaining a relevant history by asking simple questions regarding nocturnal eating may lead to more targeted and expeditious treatment. As with any disease or condition, proper therapy depends on accurate diagnosis. Moreover, these patients can be challenging and often require a multidisciplinary approach, such as collaboration with eating disorder centers, psychiatrists and other behavioral professionals, and sleep medicine specialists.

The patient in the present case demonstrated a clear temporal association between cessation of SRED behaviors and initiation of PHEN-TPM ER. This is the first reported case of successful treatment of SRED using the novel anti-obesity agent PHEN-TPM ER. Therapeutic options are limited. This report demonstrates a potential new tool and will hopefully trigger more research in this area. However, there are some limitations to this case report that should be considered. At presentation, the patient reported excessive alcohol consumption on the weekends. Although he reported significant improvement on follow-up, it is unclear if this may have been causal in improving quality of sleep as well. Furthermore, it is unknown to what degree his underlying OSA may have contributed to his arousals and disordered nocturnal eating behavior. Weight loss at 5 months of 5% was modest, but this is considered clinically meaningful because many comorbidities are improved with this degree of weight reduction [15]. It is also unclear if this patient would have lost the same amount of weight with topiramate monotherapy. Whether the patient may have lost more weight with more intense lifestyle interventions, dose escalation, or longer term therapy is unknown.

SRED is not a common disorder but given the ubiquity of obesity and related comorbidities in general clinical practice, the overall prevalence is likely not trivial and proper identification of these patients would likely improve their outcomes. Although further studies are needed to determine if dual use of phentermine and topiramate is more efficacious than topiramate monotherapy for the treatment of SRED, clinicians should consider this therapeutic combination for appropriate patients presenting with both SRED and obesity.

Diagnostic criteria for sleep-related eating disorder

**Table:** 

A. Recurrent episodes of involuntary eating and drinking during the main sleep period
B. One or more of the following must be present with these recurrent episodes:
1. Consumption of peculiar, inedible, or toxic substances
2. Insomnia related to repeated episodes of eating, with a complaint of nonrestorative sleep, daytime fatigue, or somnolence
3. Sleep-related injury
4. Dangerous behavior while preparing food
5. Morning anorexia
6. Adverse health consequences from recurrent binge eating
C. The disturbance is not better explained by another sleep, medical, or neuropsychiatric disorder

Adapted from Howell (2007) [5]

## Conclusions

Obesity is one of the most common chronic diseases encountered in clinical practice. SRED is uncommon and is rarely diagnosed or considered when assessing patients with excess weight. Presence of eating disorders should be screened for when evaluating a patient for weight reduction strategies. Both are challenging to manage. When diagnosed as comorbid conditions, healthcare professionals should consider the use of PHEN-TPM ER as dual therapy.

## Data Availability

All data generated or analyzed during this study are included in this published article.

## Abbreviations

AOM: Anti-obesity medication
BED: Binge eating disorder
BMI: Body mass index
CNS: Central nervous system
NES: Night eating syndrome
OSA: Obstructive sleep apnea
PHEN-TPM ER: Phentermine–topiramate extended release
RLS: Restless leg syndrome
SRED: Sleep-related eating disorder
US FDA: Unites States Food and Drug Administration
